# Fine mapping of *Rha2* in barley reveals candidate genes for resistance against cereal cyst nematode

**DOI:** 10.1007/s00122-019-03279-3

**Published:** 2019-01-18

**Authors:** Bart Van Gansbeke, Kelvin H. P. Khoo, John G. Lewis, Kenneth J. Chalmers, Diane E. Mather

**Affiliations:** 1 School of Agriculture, Food and Wine, Waite Research Institute, The University of Adelaide, PMB 1, Glen Osmond, SA 5064 Australia; 2 South Australian Research and Development Institute, GPO Box 397, Adelaide, SA 5001 Australia

## Abstract

**Key message:**

The cereal cyst nematode resistance locus *Rha2* was mapped to a 978 kbp region on the long arm of barley chromosome 2H. Three candidate genes are discussed.

**Abstract:**

The cereal cyst nematode (CCN) *Heterodera avenae* is a soil-borne obligate parasite that can cause severe damage to cereals. This research involved fine mapping of *Rha2*, a CCN resistance locus on chromosome 2H of barley. *Rha2* was previously mapped relative to restriction fragment length polymorphisms (RFLPs) in two mapping populations. Anchoring of flanking RFLP clone sequences to the barley genome assembly defined an interval of 5077 kbp. Genotyping-by-sequencing of resistant and susceptible materials led to the discovery of potentially useful single nucleotide polymorphisms (SNPs). Assays were designed for these SNPs and applied to mapping populations. This narrowed the region of interest to 3966 kbp. Further fine mapping was pursued by crossing and backcrossing the resistant cultivar Sloop SA to its susceptible ancestor Sloop. Evaluation of F_2_ progeny confirmed that the resistance segregates as a single dominant gene. Genotyping of 9003 BC_2_F_2_ progeny identified recombinants. Evaluation of recombinant BC_2_F_3_ progeny narrowed the region of interest to 978 kbp. Two of the SNPs within this region proved to be diagnostic of CCN resistance across a wide range of barley germplasm. Fluorescence-based and gel-based assays were developed for these SNPs for use in marker-assisted selection. Within the candidate region of the reference genome, there are nine high-confidence predicted genes. Three of these, one that encodes RAR1 (a cysteine- and histidine-rich domain-containing protein), one that is predicted to encode an acetylglutamate kinase and one that is predicted to encode a tonoplast intrinsic protein, are discussed as candidate genes for CCN resistance.

**Electronic supplementary material:**

The online version of this article (10.1007/s00122-019-03279-3) contains supplementary material, which is available to authorized users.

## Introduction

The cereal cyst nematode (CCN) *Heterodera avenae* is a soil-borne parasite that infects the roots of many grass species and can cause significant yield losses in cereal crops including barley (*Hordeum vulgare* L.), wheat (*Triticum aestivum* L.) and oat (*Avena sativa* L.). Like other cyst nematodes, it is a sedentary endoparasite. Motile J2-stage larvae enter the elongation zones of roots and migrate intracellularly through the root cortex. Upon reaching the vascular cylinder, they establish feeding sites. Initially, each feeding site consists of a single plant cell, but the nematode induces the dissolution of cell walls and adjacent cells are ‘recruited’, resulting in the formation of a multinuclear syncytium. Once the nematodes differentiate into males and females, the males detach from their feeding sites and leave the roots. The females remain within the root, growing and maturing, until their bodies become egg-filled white cysts that protrude from the roots and finally hard brown cysts within which the eggs can withstand unfavourable conditions in the soil.

Within host plant species that are affected by cyst nematodes, there is genetic variation for resistance (the ability to reduce nematode populations in the soil). Genes for resistance against cyst nematodes have been isolated from dicot species (Cai et al. 1997; Liu et al. 2012, 2017; Paal et al. 2004; van der Vossen et al. 2000), but not from any monocot species. In barley (reviewed below) and in wheat (reviewed by Jayatilake et al. 2015), resistance loci have been genetically mapped and resistance alleles have been used in cereal breeding. In Australia, where there is thought to be only one pathotype (Ha13) of *H. avenae*, consistent use of resistant cereal cultivars and cultural management practices has been very effective in reducing nematode populations in agricultural soils and preventing yield losses (Murray and Brennan 2010).

In barley, the inheritance of CCN resistance was first investigated by Nilsson-Ehle (1920), who reported results indicating segregation of a single unit of inheritance, for which resistance was dominant. Subsequent research established that there are up to three CCN resistance loci on chromosome 2H (Andersen and Andersen 1968, 1973; Cotten and Hayes 1969; Kretschmer et al. 1997). Of these, two (originally called *Ha2* and *Ha3* but now called *Rha2* and *Rha3*) are very closely linked with each other (or may even be the same locus). The other locus, *Rha1* (formerly *Ha* or *Ha1*) is not closely linked with *Rha2* and *Rha3.* There is also a CCN resistance locus (*Ha4*, now *Rha4*) on chromosome 5H (Barr et al. 1998).

The research reported here focuses on a locus that Kretschmer et al. (1997) mapped relative to molecular markers using two populations of doubled haploid (DH) barley lines: one derived from a cross between Chebec (a resistant cultivar that inherited its resistance from the Algerian cultivar Orge Martin) and Harrington (a susceptible cultivar) and one derived from a cross between Sahara 3771 (a resistant North African landrace) and Clipper (a susceptible cultivar). When these materials were inoculated with juvenile nematodes of the Ha13 pathotype of *H. avenae*, most individual lines could be unambiguously classified as resistant or susceptible. In each population, a resistance locus was mapped on the long arm of chromosome 2H, in the interval between the restriction fragment length polymorphism (RFLP) markers awbma21 and mwg694. Kretschmer et al. (1997) considered this locus to be the same as the one that confers resistance in *Hordeum pallidum* var. 191 and called it ‘*Ha 2’*. Here, we refer to it as *Rha2*. Using marker-assisted selection, breeding programs in Australia developed two CCN-resistant derivatives of the CCN-susceptible malting barley cultivar Sloop: Sloop SA (Chebec/3*Sloop) and Sloop VIC (Sahara 3771/WI2723//Chebec///2*Sloop) (Andrew Barr and David Moody, personal communication). Simple sequence repeat (SSR) markers were added to genetic maps (Barr et al. 2003; Karakousis et al. 2003; Williams et al. 2006). While some of these markers were used for selection, they were not entirely diagnostic of resistance. To overcome some of the limitations of RFLP and SSR markers for use in marker-assisted selection, Dayteg et al. (2008) developed a co-dominant sequence characterised amplified region (SCAR) marker, Ha2S18. Using a population derived from a cross between the cultivars SW Buddy and SW Cecilia, they mapped this marker on chromosome 2H and reported it to be 4.3 cM distal to *Ha2*.

With the availability of a reference assembly of the barley genome (Mascher et al. 2017), many markers that are associated with known sequences can now be anchored to specific physical positions on pseudomolecule sequences. Further, with current technologies for DNA sequencing and marker genotyping, new DNA polymorphisms can be readily discovered and mapped. In the research reported here, *Rha2*-linked markers were anchored to the 2H pseudomolecule sequence, genotyping-by-sequencing (GBS) was applied to discover informative single nucleotide polymorphisms (SNPs), and Kompetitive Allele-Specific PCR™ (KASP) technology was used for fine mapping.

## Materials and methods

### Barley materials

The plant materials used here included two populations of doubled haploid lines (Chebec/Harrington (C/H) and Clipper/Sahara 3771 (C/S)), their parents (Chebec, Harrington, Clipper and Sahara 3771), the susceptible cultivar Schooner, accessions of 17 barley lines that had previously been used to discriminate among pathotypes of *H. avenae* (Table S1 in Online Resource 1) (Andersen and Andersen 1982; Kort et al. 1964; O’Brien and Fisher 1977, 1979; Smiley et al. 2011) and 175 other cultivars of barley (Table S2 in Online Resource 1). The C/H and C/S populations were the same as those used by Kretschmer et al. (1997).

**Fig. 1:**
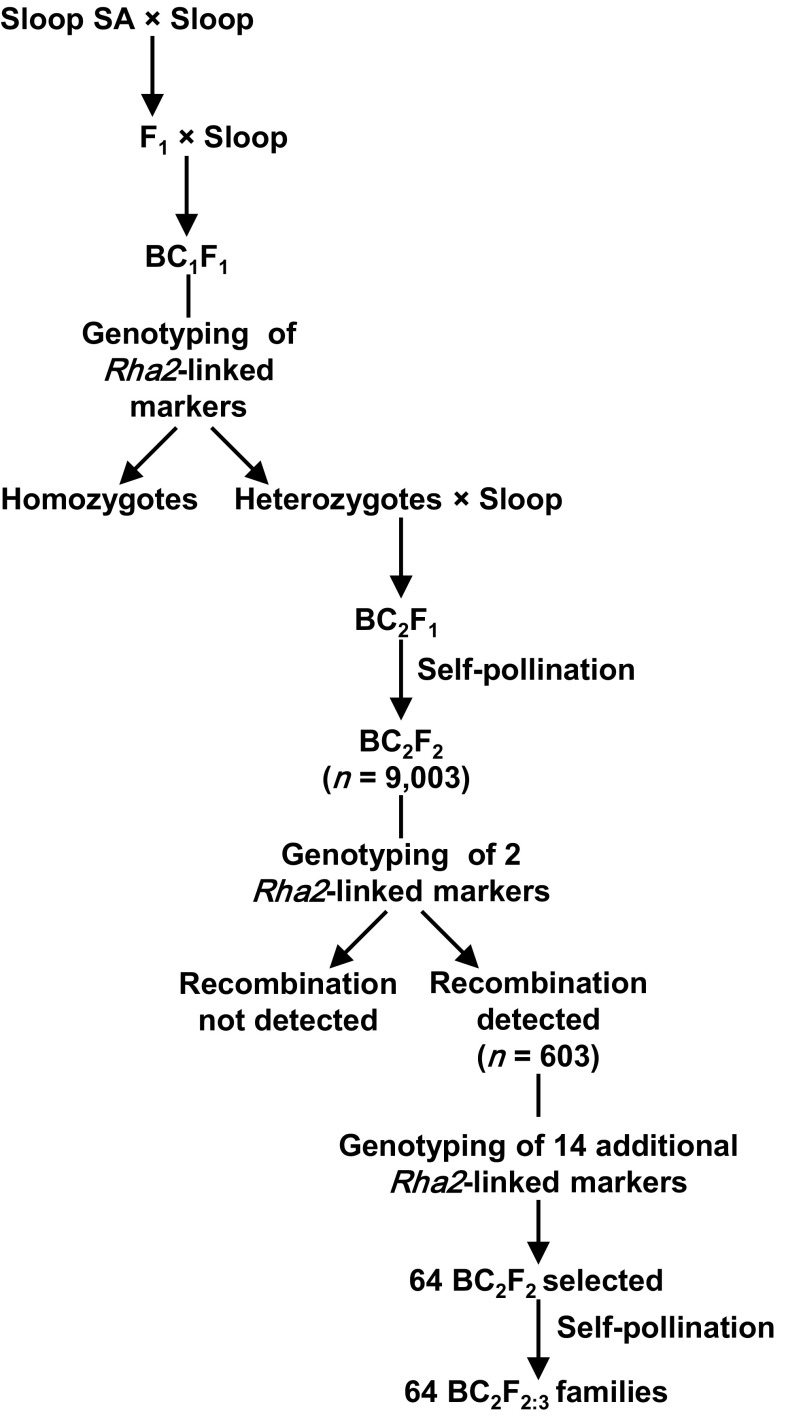
Marker-assisted backcrossing scheme used to generate BC_2_F_2:3_ families segregating for recombinant haplotypes in the *Rha2* region of chromosome 2H

To develop additional materials for this research, Sloop SA was crossed with Sloop, F_1_ plants were grown and allowed to self-pollinate, and F_2_ seeds were harvested at maturity. Some Sloop SA/Sloop F_1_ plants were backcrossed to Sloop, providing BC_1_F_1_ progeny (Fig. 1). Selected BC_1_F_1_ progeny were backcrossed to Sloop, providing BC_2_F_1_ progeny. These plants were grown to maturity, providing 9003 BC_2_F_2_ seeds.

### Methods for evaluation of resistance against cereal cyst nematode

Two methods were used to evaluate the resistance of barley plants against CCN: ‘tube tests’ and ‘pots tests’ (Figures S1 to S8 in Online Resource 2). For tube tests (Fisher 1982; O’Brien and Fisher 1977), opaque plastic tubes (2.5 cm internal diameter and 13 cm deep) were filled with pre-sterilised sandy loam soil and set upright in a base of potting mixture. One pre-germinated seed was sown in each tube, and the tubes were transferred to a controlled environment room that was maintained at a constant temperature of 15 °C with a 12 h light/dark cycle. At 1, 4, 7, 11 and 14 days after sowing, 1 mL of inoculum (a suspension of 100 J2-stage *H. avenae* larvae per mL water) was pipetted onto the soil surface around each seedling. At 70 d after the final inoculation, the soil was washed off the roots and white *H. avenae* females (white cysts) were counted. For pots tests (Lewis et al. 2009), pots (5 cm in diameter and 10 cm deep) were filled with a mixture of sandy loam soil and Osmocote Plus 8–9 Month slow release fertiliser (ICL-Specialty Fertilizers, The Netherlands) that was infested with mature *H. avenae* cysts to provide approximately 25 eggs per g of soil. Pots were arranged in wire mesh crates (50 pots per crate in a 5 × 10 array). One seed was sown in each pot. The crates were placed outdoors in autumn, on well-drained terraces in Urrbrae, South Australia (34°58′9.5″S 138°38′25.0″). Supplementary irrigation was provided as needed. At 84 d after sowing, the root balls were removed from the pots. The number of white cysts on the surface of each root ball was counted.

While tube tests can be conducted at any time of year, and they are generally considered to be more reliable and precise than pots tests, they are more expensive to conduct and their throughput is limited by the size and availability of controlled environment facilities. In Australia, tube tests are used for official classification of varieties for CCN resistance, while pots tests have been used for preliminary screening of breeding lines. In our experience, the resistance conferred by *Rha2* can be readily detected in either type of test. The research described here involved evaluation of resistance in successive generations over several years. At each stage, the choice to use tube tests or pots tests was made based on practical considerations including time of year, availability of facilities, cost and the numbers of plants to be evaluated. In both types of test, white cysts are counted for each individual plant. If the count is high, that plant can be unambiguously scored as susceptible. However, if the count is low, it is not possible to be sure that the plant is resistant, as it could be a susceptible plant that ‘escaped’ infection. Therefore, it is important to use replication. For evaluation of the resistance status of cultivars and other accessions, between four and ten replicates (plants) of each accession were included in tube tests. For fine mapping, replication was achieved by evaluating multiple progeny of recombinant plants, with at least 16 plants evaluated for each recombinant haplotype.

### Determination of the physical positions of RFLP and SCAR markers

Clone sequences for five RFLP markers (mwg892, mwg865, psr901, awbma21 and mwg694) that had been mapped by Kretschmer et al. (1997) and the sequence for which Dayteg et al. (2008) had developed the SCAR marker Ha2S18 were subjected to a BLASTn analysis (Altschul et al. 1990) against the International Barley Genome Sequencing Consortium 2H pseudomolecule (150831_barley_pseudomolecules_chr2H.fasta, downloaded from http://webblast.ipk-gatersleben.de/barley_ibsc/downloads/) (Mascher et al. 2017).

### Evaluation of mapping data

For the C/H and C/S populations, genotypic and phenotypic data that had been used by Kretschmer et al. (1997) were examined. Subsets of lines with parental haplotypes in the region of interest and for which the existing phenotypic data were consistent with the observed haplotype (i.e. few or no white cysts for lines with the Chebec or Sahara 3771 haplotype; high numbers of white cysts for lines with the Harrington or Clipper haplotype) were selected for use in genotyping-by-sequencing to discover DNA polymorphisms. Lines that had recombinant haplotypes in the region of interest but for which the existing phenotypic data were missing or inconclusive were evaluated in tube tests to confirm their resistance status.

### Genotyping-by-sequencing

The genomic DNA used for GBS analysis included samples isolated from leaf tissue sampled from one plant of each of Chebec, Harrington, Clipper, Sahara 3771, selected C/H and C/S DH lines (some resistant and some susceptible), the resistant barley cultivars Sloop SA and Sloop VIC and 13 susceptible barley cultivars (Baudin, Buloke, Cowabbie, Fitzroy, Gairdner, Hamelin, Malloy, Oxford, Skiff, Schooner, Sloop, Tantangara and Vlamingh). For these samples, DNA was isolated from milled leaf samples using a phenol chloroform method (Rogowsky et al. 1991) with modifications as described by Pallotta et al. (2000). Five pooled DNA samples were formed by mixing equal quantities of DNA of each member of five sets of lines: 66 resistant C/H lines with the Chebec haplotype in the region of interest on chromosome 2H; 22 susceptible C/H lines with the Harrington haplotype in the region of interest; 71 resistant C/S lines with the Sahara 3771 haplotype in the region of interest; 71 susceptible C/S lines with the Clipper haplotype in the region of interest on chromosome 2H; and 12 of the 13 susceptible cultivars (all except Sloop). One aliquot for each of Sloop, Sloop SA, Sloop VIC, Chebec, Harrington, Clipper and Sahara 3771 and one aliquot of each pooled sample were sent to Diversity Arrays Technology (Bruce, ACT, Australia) for analysis with its DArTseq GBS platform (www.diversityarrays.com/dart-application-dartseq).

The GBS sequence data were analysed by Diversity Arrays Technology using its proprietary software pipeline. Tag sequences that were reported as including SNPs were used in BLASTn analysis (Altschul et al. 1990) against the 2H pseudomolecule of the barley reference genome (Mascher et al. 2017). Tags with a BLAST hit alignment (minimum e-value = 1e^−5^) were selected.

To select SNPs that segregate in the C/H and/or C/S populations and that would map in the region of interest, results for contrasting (resistant and susceptible) pools were compared with each other and with the results for their resistant and susceptible parents. To identify SNPs that could be widely applicable in marker-assisted selection for CCN resistance, the selected tag pairs were filtered to retain only those for which Sahara 3771, Chebec and both pools of resistant lines exhibited only one tag and for which Clipper, Harrington and all pools of susceptible lines or cultivars exhibited only the alternate tag.

### Development and application of marker assays

Primers for KASP marker assays (Table S3 in Online Resource 1) were designed for selected SNPs using Kraken™ software (LGC Genomics Limited, Hoddlesdon, UK). The resulting assays were applied using an automated SNPLine™ system (LGC Genomics Limited, Hoddlesdon, UK) according to the manufacturer’s instructions. Some of the DNA samples used for this were isolated from leaf tissue sampled from young seedlings. Other DNA samples were isolated from endosperm tissue, so that individual seeds could be genotyped to select those to be germinated for evaluation of resistance. Endosperm tissue samples were obtained by dissecting individual barley grains into two parts: one containing the embryo and the other consisting mostly of endosperm. Detailed protocols for tissue sampling, tissue preparation and DNA extraction are given in Online Resource 3.

To provide gel-based assays for two SNPs that were determined to be very closely linked to *Rha2*, temperature-switch PCR primer sets (Table 1) were designed using the methods described by Tabone et al. (2009). Briefly, primers were designed with Primer3 release 2.3.7 (Rozen and Skaletsky 2000) by using genomic sequence retrieved from the 2H pseudomolecule. Each assay consists of a locus-specific primer pair and a nested allele-specific primer. The assays were performed with a QIAGEN Taq DNA polymerase kit in a 10 μl reaction mixture containing 2 μl template DNA (10 ng/µl), 0.1 μl polymerase, 1 μl 10 × buffer, 1.6 μl dNTPs (1.25 mM), 2 μl 5 × Q solution, 0.1 μl locus-specific forward primer (10 μM), 0.1 μl locus-specific reverse primer (10 μM), 0.5 μl nested allele-specific primer (10 μM) and 2.6 μl sterile nuclease-free water. The amplification protocol was as follows: (1) an initial denaturation step of 10 min at 95 °C, (2) 15 cycles of 30 s at 94 °C, 30 s at 58 °C and 60 s at 72 °C, (3) 8 cycles of 10 s at 94 °C and 30 s at 45 °C, (4) 15 cycles of 30 s at 94 °C, 30 s at 53 °C and 30 s at 72 °C. Finally, the samples were cooled to 20 °C. The reactions were run on DNA Engine Dyad Peltier Thermal Cycler (Bio-Rad Laboratories, Hercules, California, USA). The PCR products were separated on a 1.5% (w/v) agarose gel containing SYBR™ Safe gel stain (Life Technologies Australia Pty Ltd., Mulgrave, VIC, Australia) for 45 min at 110 V and were visualised with a UV transilluminator.

**Table 1:** Primer sequences for temperature-switch PCR assays wri328 and wri329, which were designed to assay the same SNPs as KASP assays wri321 and wri297, respectively

Primer	wri328	wri329
LSF1	AGGTGATCACGATCTCCATCACCAC	GCGGATGCAATGGAGGTCTA
LSR1	CTTCTTGTGCAGGGCAACTGAC	ACGGAATGCTCCCCTAGGAA
ASF1	GGAAACTGCAGGAGGAA	GTGAGATGCAATTGAAATCG

## Results

Phenotypic evaluation of a small panel of accessions in a tube test showed that Athinais, Bajo Aragon, Barley 191, Martin 403-2, Morocco, Morocco (Early), Nile, Orge Martin, Orge Martin 839, Sabarlis and Siri are all resistant to the *H. avenae* pathotype that was used here, with mean numbers of white cysts ranging from 0 to 1.1 per plant (Table S1 in Online Resource 1) while Alfa, Drost, Herta, Ortolan, Schooner and Varde are all susceptible to that pathotype, with mean numbers of white cysts ranging from 5.4 to 10.7 per plant. Phenotypic results for the available accessions of Marocaine 079 and Quinn were inconclusive.

Sequences associated with five RFLP markers that Kretschmer et al. (1997) had mapped near the resistance locus were anchored to the pseudomolecule sequence for chromosome 2H at positions between 654,782 (mwg865) and 684,123 kbp (mwg694) (Table S4 in Online Resource 1). The sequence for which Dayteg et al. (2008) had developed a SCAR marker (Ha2S18) was anchored just distal to these positions, at 685,898 kbp.

**Fig. 2:**
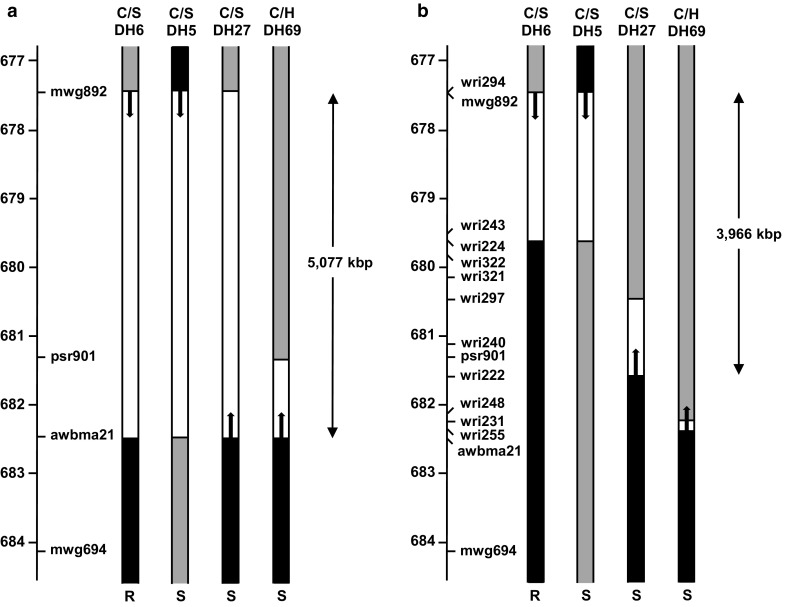
Graphical representation of *Rha2*-region genotypes of three Clipper/Sahara 3771 (C/S) doubled haploid lines and one Chebec/Harrington (C/H) doubled haploid line. In each case, the axis to the left of the graphical genotypes shows the physical positions of markers (in Mbp) on the 2H pseudomolecule of the barley genome assembly. **a** Graphical genotypes based on pre-existing RFLP marker information. **b** Graphical genotypes based on both pre-existing RFLP marker information and new KASP marker information. In each graphical genotype, the region shaded in black was inherited from the resistant parent (Sahara 3771 or Chebec), the region shaded in grey was inherited from the susceptible parent (Harrington or Clipper), and the unshaded region is the region within which recombination occurred. The cereal cyst nematode resistance status of each line is indicated by the letter R (resistant) or S (susceptible) at the bottom of the figure. Single-headed arrows point in the direction towards which the resistance locus can be deduced to lie based on the genotype and phenotype of each individual line. Double-headed arrows define the candidate intervals for *Rha2* based on this information

With examination of RFLP data for the C/H and C/S populations, four DH lines were identified as having recombination events between mwg892 (677,498 kbp) and awbma21 (682,575 kbp): C/S DH5, C/S DH6, C/S DH27 and C/H DH69. Phenotypic and genotypic data for two of these lines (C/S DH5 and C/S DH6) indicate that the resistance locus is distal to mwg892, while the data for the other two lines (C/S DH27 and C/H DH69) indicate that the resistance locus is proximal to awbma21 (Fig. 2a). Based on these observations, the 5077 kbp region between mwg892 and awbma21 was considered as the candidate region for *Rha2*.

**Fig. 3:**
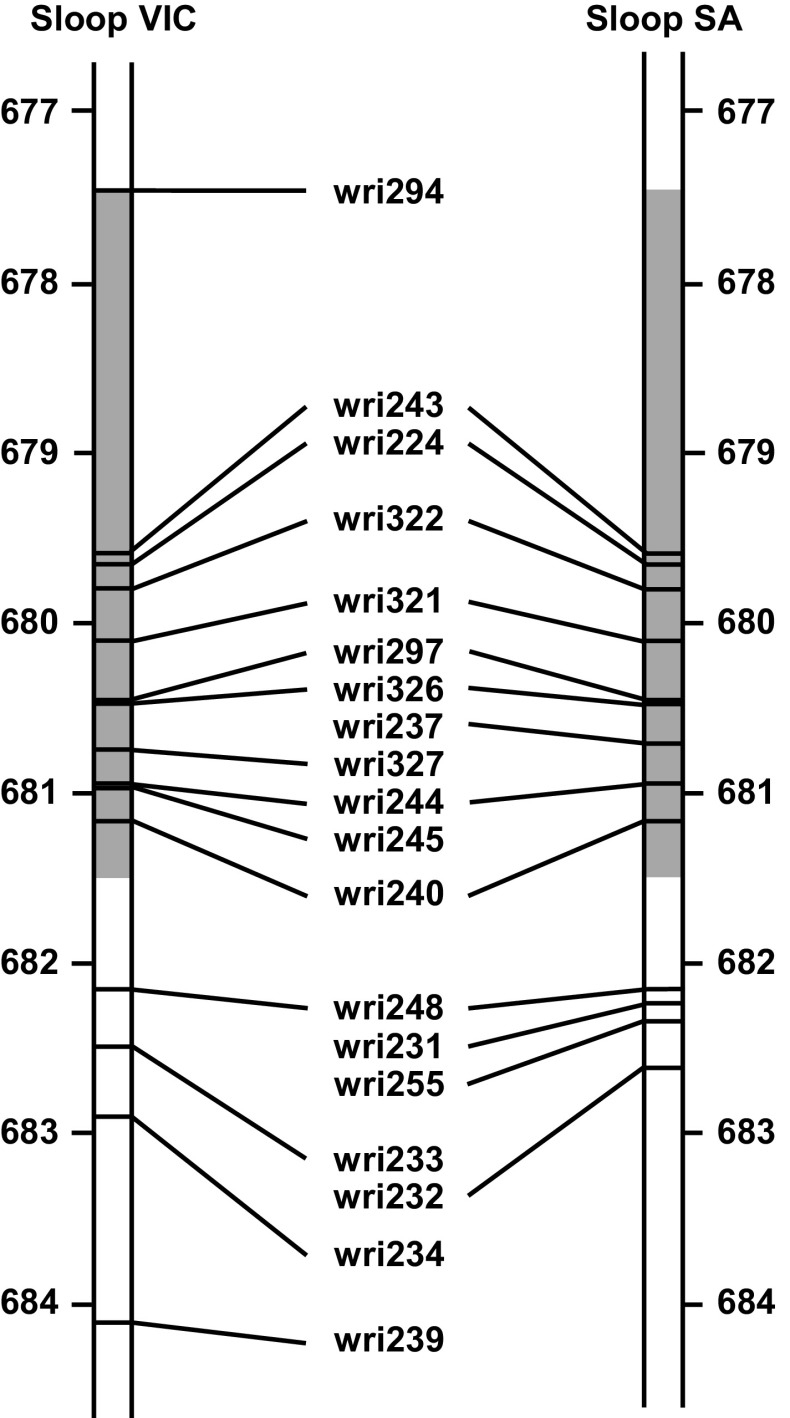
A candidate region (shaded) for *Rha2*-on barley chromosome 2H, showing the physical positions (in Mbp on the 2H pseudomolecule of the barley genome assembly) at which KASP marker assays revealed single nucleotide polymorphisms between the susceptible cultivar Sloop and one or both of its resistant derivatives Sloop VIC (left) and Sloop SA (right)

Analysis of the GBS data yielded 8923 SNP-bearing tag pairs, of which 1937 could be anchored to the pseudomolecule sequence for chromosome 2H (Table S5 in Online Resource 1). Of these, 38 were anchored within the candidate region (677,498–682,575 kbp). KASP assays were designed for 106 SNPs, including 24 in the candidate region. With application of 11 KASP assays to the four recombinant DH lines (C/S DH5, C/S DH6, C/S DH27 and C/H DH69), the candidate region for *Rha2* was narrowed to the 3966 kbp region between mwg892 (677,498 kbp) and wri222 (681,464 kbp) (Fig. 2b). Consistent with this, application of KASP assays to Sloop, Sloop VIC and Sloop SA, indicated that Sloop VIC and Sloop SA each differ from Sloop in the candidate region (Fig. 3). For six consecutive markers (wri243, wri224, wr322, wri321, wri297 and wri326) there were no genotype differences detected between Sloop VIC and Sloop SA, indicating that the region from 679,677 to 680,443 kbp could be identical-by-descent in these two resistant cultivars.

**Fig. 4:**
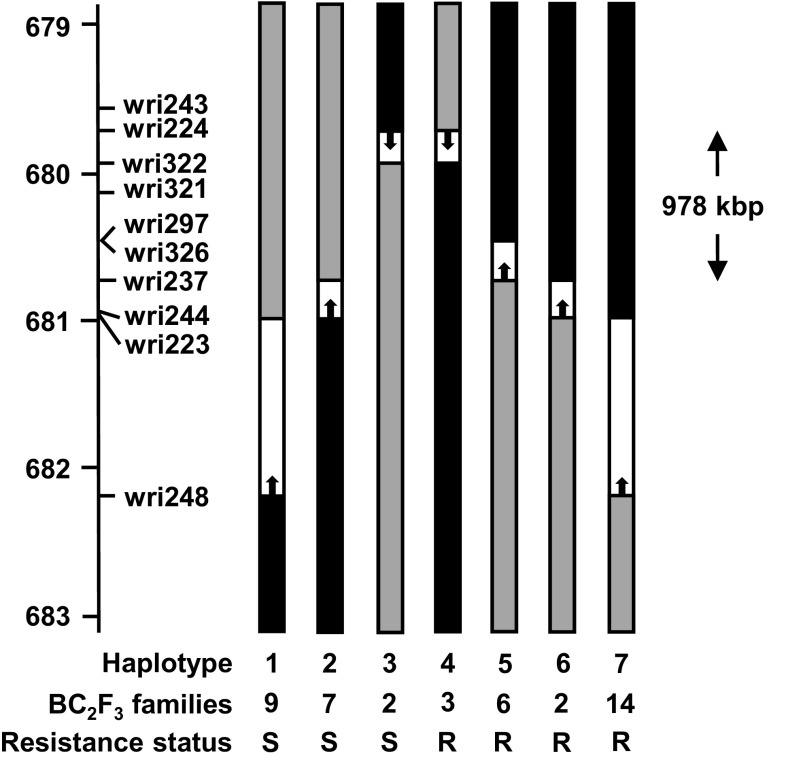
Graphical representations of seven recombinant *Rha2*-region haplotypes observed in progeny derived by backcrossing the resistant cultivar Sloop SA to its susceptible parent Sloop. The axis to the left of the graphical genotype shows the physical positions of markers (in Mbp) on the 2H pseudomolecule of the barley genome assembly. In each graphical genotype, the region shaded in black was inherited from the resistant parent (Sloop SA), the region shaded in grey was inherited from the susceptible parent (Sloop) and the unshaded region is the region within which recombination occurred. For each haplotype, the number of BC_2_F_3_ families assessed is shown and the resistance status of those families is indicated as R (resistant) or S (susceptible). The double-headed arrow defines a candidate interval for *Rha2* based on this information

**Fig. 5:**
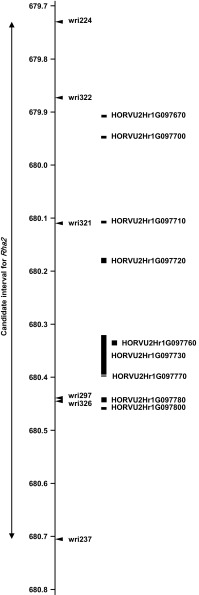
Positions of six single nucleotide polymorphisms (indicated by triangles and labelled by the names of KASP marker assays) and nine high-confidence predicted genes (rectangles) relative to a 978 kbp candidate interval for *Rha2* on the 2H pseudomolecule of the barley genome assembly

KASP assays for wri243 (679,677 kbp) and wri256 (685,490 kbp) were applied to 9003 BC_2_F_2_ progeny of Sloop and Sloop SA. Based on the results obtained, 603 plants carrying recombinant haplotypes were selected for use in fine mapping. Co-dominant KASP assays for 14 additional markers were applied to the 603 selected plants. The results provided a genetic order that was consistent with the physical order of the markers on the 2H pseudomolecule, with only two exceptions: (1) wri240 (681,155 kbp) did not map in the region and (2) wri255 (682,309 kbp) was found to be proximal to wri231 (682,242 kbp). Sixty-four plants with recombination events between wri243 (679,677 kbp) and wri232 (682,572 kbp) were grown and allowed to self-pollinate to provide BC_2_F_3_ families. Members of 53 BC_2_F_3_ families were evaluated for CCN resistance and were genotyped (Table S6 in Online Resource 1). With comparison of the phenotypic and genotypic results, the region of interest was narrowed to the 978 kbp interval between wri224 (679,727 kbp) and wri237 (680,705 kbp) (Fig. 4). This interval coincides with the region in which Sloop VIC and Sloop SA could not be distinguished from each other. Within that region, nine genes have been predicted with high confidence (Table 2, Fig. 5) (Mascher et al. 2017).

**Table 2:** High-confidence predicted genes in the candidate region between 679,727 kbp and 680,705 kbp on chromosome 2H (Mascher et al. 2017)

Gene code	Position	Annotation
HORVU2Hr1G097670	679,904,944–679,907,205	Plastid-lipid-associated protein
HORVU2Hr1G097700	679,946,510–679,947,405	Unknown protein
HORVU2Hr1G097710	680,104,708–680,108,022	F-box family protein
HORVU2Hr1G097720	680,177,439–680,186,077	Acetylglutamate kinase
HORVU2Hr1G097730	680,321,819–680,393,065	Acetylglutamate kinase
HORVU2Hr1G097760	680,332,215–680,337,187	Uncharacterised conserved protein
HORVU2Hr1G097770	680,394,226–680,395,854	Carotenoid cleavage dioxygenase 7
HORVU2Hr1G097780	680,440,771–680,446,606	Aquaporin-like superfamily protein
HORVU2Hr1G097800	680,457,446–680,461,502	Cysteine- and histidine-rich domain-containing protein, RAR1

Among all progeny to which the assays for wri322 (679,878 kbp), wri321 (680,107 kbp), wri297 (680,441 kbp) and wri326 (680,443 kbp) were applied, no recombination was observed among these markers. Among 101 Sloop SA/Sloop F_2_ plants that were evaluated in a pot test, the segregation ratio observed for these markers was 20:52:29 (Sloop SA homozygotes/heterozygotes/Sloop homozygotes), which does not deviate significantly from the expected 1:2:1 ratio (Chi-square: 1.69; *p *= 0.43; *df* = 2). Of the 72 plants with Sloop SA alleles in either the homozygous or heterozygous state, none had more than three white cysts (Table S7 in Online Resource 1). Similar results were obtained for Sloop SA control plants. Among the other 29 plants (Sloop homozygotes), numbers of white cysts ranged from 0 to 22. A wide range (0–34 white cysts) was also observed among Sloop control plants. Overall, these results are consistent with the resistance of Sloop SA being conferred by a single dominant gene that is closely linked with wri322, wri321, wri297 and wri326.

When the assays for the four genetically co-segregating markers (wri322, wri321, wri297 and wri326) were applied to accessions of barley lines that have previously been used to differentiate among pathotypes of *H. avenae*, five haplotypes were observed (Table S1 in Online Resource 1). As expected, the resistance-associated T-A-A-G haplotype that is present in Chebec was also observed for its resistant ancestor Orge Martin. This haplotype was also observed for Bajo Aragon, Barley 191, Morocco, Martin 403-2, Orge Martin 839, Sabarlis and Siri, all of which have been classified as carrying *Rha2* and/or *Rha3* (Andersen and Andersen 1982; Smiley et al. 2011). Four other haplotypes were observed: two in accessions that were found to be resistant (C-G-G-G in Morocco (Early) and Athinais, and C-A-A-G in Nile) and two (C-G-G-A and C-G-G-null) among cultivars that were found to be susceptible (Alfa, Clipper, Drost, Herta, Ortolan, Varde and Schooner). For each of Quinn and Marocaine 079, the two accessions evaluated exhibit different haplotypes: (C-G-G-null and C-G-G-A for Quinn; T-A-A-G and C-G-G-A for Marocaine 079).

Assays for the four co-segregating markers (wri322, wri321, wri297 and wri326) were applied to a large panel of barley cultivars. The results (Table S2 in Online Resource 1) indicate that the wri326 assay is neither reliable nor diagnostic of resistance. With this assay, some samples were readily called as G:G homozygotes (predominantly HEX fluorescence) or A:A homozygotes (predominantly FAM fluorescence). Others were called as ‘nulls’ because little fluorescence of either type was detected. Still others could not be called because their results were intermediate (between the null and A:A clusters or between the G:G and A:A clusters). With the susceptible cultivars Brindabella, Clipper and Harrington all exhibiting the resistance-associated G:G genotype, wri326 is clearly not diagnostic of resistance. This marker was therefore excluded from further consideration for use in barley breeding. With assays wri322, wri321 and wri297, four haplotypes were observed: T-A-A, C-G-G, null-G-G and T-G-G. The resistance-associated T-A-A haplotype was detected for the resistant parents Chebec and Sahara 3771, their resistant derivatives Sloop SA and Sloop VIC, two other resistant cultivars (Dash and Hindmarsh) and seven cultivars of unknown resistance status (Albacete, Alf, Fractal, GrangeR, Harbin, Optic and SY Rattler). The opposite (C-G-G) haplotype was detected for 31 susceptible cultivars, 16 resistant cultivars whose resistance is due to the *Rha4* locus that Barr et al. (1998) mapped on chromosome 5H (Barque, Capstan, Commander, Doolup, Dhow, Fathom, Flagship, Fleet Australia, Galleon, Keel, Maritime, Navigator, Skipper, Torrens and Yarra) and 112 cultivars of unknown resistance status. The T-G-G haplotype was observed for Brindabella and Haruna Nijo (both known to be susceptible) and for Digger, Kearney and Prior Early (all with unknown resistance status). The null-G-G haplotype was detected for Azumamugi, Kikkaihadaka and Zavilla (all with unknown resistance status). When the T-A-A, T-G-G and null-G-G cultivars of unknown resistance status were compared to Chebec (resistant; T-A-A) and Schooner (susceptible; C-G-G) in a tube test, all seven T-A-A cultivars were classified as resistant (with mean numbers of white cysts between 0 and 4.5 per plant) and all T-G-G and null-G-G cultivars were classified as susceptible (with mean numbers of white cysts between 32.8 and 54.3 per plant) (Table S8 in Online Resource 1).

For the A > G SNPs assayed by wri297 and wri321, additional assays (wri328 and wri329, respectively), were developed to make it possible to assay the target SNPs using temperature-switch PCR (Tabone et al. 2009) and gel electrophoresis. With each of these assays (Table 1), one product (457 bp for wri328 and 335 bp for wri329) was amplified when the susceptibility-associated nucleotide G was present and a product of a different length (250 bp for wri328; 514 bp for wri329) was amplified when the resistance-associated nucleotide A was present (Fig. S9 in Online Resource 2). For heterozygous samples, both products were visible for each assay.

## Discussion

It is generally accepted that Ha13 is the prevalent pathotype of *H. avenae* in Australia (Brown 1982) and it is known that CCN resistance derived from either Chebec or Sahara 3771 is effective in Australia. Kretschmer et al. (1997) attributed the resistance of both Chebec and Sahara 3771 to the *Rha2* locus on chromosome 2H. According to Smiley et al. (2011), *Rha3* confers resistance against the Ha13 pathotype. This led us to question whether Chebec and Sahara 3771 might carry *Rha3* rather than *Rha2*. To investigate this, we evaluated materials that had previously been reported to carry *Rha2* and/or *Rha3* resistance. They all exhibited resistance against the pathotype used in this research. As we could not differentiate between *Rha2* and *Rha3* materials either phenotypically or genotypically, we retained the designation *Rha2* for the locus mapped by Kretschmer et al. (1997), even though we could not unequivocally demonstrate that this resistance is identical-by-descent with that of Barley 191, which was the original source of *Rha2* investigated by Andersen and Andersen (1968).

The analysis conducted here for two mapping populations demonstrates that the use of pre-existing data in combination with current genome sequence information (Mascher et al. 2017) can help define the physical position of a locus that was previously only roughly mapped relative to RFLP markers. With this approach, it was possible to define a 5077 kbp region of the chromosome 2H pseudomolecule as the candidate region for *Rha2*. With the application of DArTseq GBS technology to bulks of resistant and susceptible mapping lines and with the anchoring of GBS tag sequences to the barley genome assembly, informative SNPs were discovered in that region. With KASP genotyping of SNPs on recombinant lines from the mapping populations, the region of interest was narrowed to 3966 kbp. Consistent with this, the resistant cultivars Sloop VIC and Sloop SA were both found to differ from their susceptible ancestor Sloop at markers within the region of interest. For Sloop SA, Chebec is the only possible source of resistance. For Sloop VIC, the source of resistance is a less clear, given that Sloop VIC has both Sahara 3771 and Chebec in its pedigree. Based on results obtained using the wri294 assay for a SNP at 677,483,533 kbp (A for Sloop, Chebec and Sloop SA; G for Sahara 3771 and Sloop VIC) and the wri327 assay for a SNP at 680,719,172 (G for Sloop, Chebec and Sloop SA; C for Sahara 3771 and Sloop VIC), it seems likely that the Sloop VIC *Rha2* segment originated from Sahara 3771.

Further narrowing of the region required new progeny with recombinant haplotypes in the region and new molecular markers to distinguish among haplotypes. Therefore, a large set of BC_2_F_2_ progeny was generated and screened with KASP assays for SNPs that had been discovered by GBS. With genotypic and phenotypic analysis of BC_2_F_3_ progeny, the region of interest was narrowed to 978 kbp. In the BC_2_F_2_ fine map, the candidate region consists of a proximal flanking marker (wri224, 679,727 kbp), a distal flanking marker (wri237, 680,705 kbp) and four co-segregating markers: wri322 (679,878 kbp), wri321 (680,107 kbp), wri297 (680,441 kbp) and wri326 (680,443 kbp). Within the 978-kbp region between the flanking markers, nine genes have been predicted with high confidence.

According to information in the BARLEX database (Colmsee et al. 2015), four of the nine high-confidence predicted genes between 679,727 and 680,705 kbp on the 2H pseudomolecule (Fig. 5), are expressed in young roots of barley. One of these (HORVU2Hr1G097760) is annotated as encoding an ‘uncharacterised conserved protein’. Conserved domain analysis for the predicted protein product did not identify any characterised functional domains. The other three (HORVU2Hr1G097800; HORVU2Hr1G097720 and HORVU2Hr1G097780) will be discussed here as possible candidates for *Rha2*.

HORVU2Hr1G097800 encodes a zinc-binding protein (RAR1; required for *Mla12* resistance) containing a highly conserved cysteine- and histidine-rich domain (CHORD, PF04968). The RAR1 protein is known to contribute to hypersensitive responses of barley against powdery mildew (*Blumeria graminis* f. sp *hordei*) (Shirasu et al. 1999). In hypersensitive responses against fungal pathogens, entry of the pathogen into resistant plants is halted at the infection site by rapid death of infected cells (Hückelhoven et al. 1999, 2000; Hückelhoven and Kogel 1998; Shirasu et al. 1999). This differs from the interactions of cyst nematodes with their hosts, in that juvenile cyst nematodes can readily invade the roots of resistant plants, migrate through cortical cells and establish feeding sites (Grymaszewska and Golinowski 1991; Holtmann et al. 2000; Seah et al. 2000; Williams and Fisher 1993; Wyss and Zunke 1986). However, shortly after the establishment of feeding sites, the affected plant cells can begin to deteriorate in resistant plants but not in susceptible plants (Endo 1991; Rice et al. 1985; Sobczak et al. 2005). The reaction observed in resistant plants has been described as a hypersensitive response resulting in a necrotic layer around the feeding cell (Grymaszewska and Golinowski 1991; Kim et al. 1987; Mahalingam and Skorupska 1996; Rice et al. 1985, 1987; Yu and Steele 1981). Consistent with this, some genes that confer cyst nematode resistance in dicot species (Cai et al. 1997; Liu et al. 2012, 2017; Paal et al. 2004; van der Vossen et al. 2000) are known to encode nucleotide-binding site leucine-rich repeat (NBS-LRR) proteins that contribute to hypersensitive responses and Lagudah et al. (1997) suggested NBS-LRR-encoding genes as candidates for the wheat *Cre3* CCN resistance locus. Thus, HORVU2Hr1G097800 seems worthy of investigation as a plausible candidate for *Rha2*.

HORVU2Hr1G097720 is annotated as encoding an acetylglutamate kinase. Acetylglutamate kinases are required for synthesis of l-arginine, which is in turn required for production of l-orthinine and the polyamines putrescine, spermidine and spermine. These polyamines have been detected at elevated levels in barley leaf tissue infected with leaf rust (*Puccinia hordei*) (Greenland and Lewis 1984) or powdery mildew (Walters et al. 1985). Research conducted with other plant species has demonstrated that spermidine and spermine play roles in plant defence. Spermidine contributes to the formation of pyrrolizidine alkaloid defence compounds (reviewed by Takahashi and Kakehi 2009; Ober and Hartmann 1999). Spermine induces accumulation of acidic pathogenesis-related (PR) proteins that are associated with hypersensitive responses (Yamakawa et al. 1998). Thus, HORVU2Hr1G097720 also seems worthy of investigation as a plausible candidate for *Rha2*.

HORVU2Hr1G097780 is annotated as encoding an aquaporin-like protein (Mascher et al. 2017). According to information in the BARLEX database (Colmsee et al. 2015), this gene exhibits root-specific expression. With comparison of the HORVU2Hr1G097780 sequence with the barley aquaporin gene family, HORVU2Hr1G097780 was identified as tonoplast intrinsic protein 2;2 (HvTIP2;2 GenBank accession number AB540223) (Hove et al. 2015). In a phylogenetic study based on major intrinsic protein sequences of the monocots barley, maize (*Zea mays*) and rice (*Oryza sativa*) and the dicot Arabidopsis (*Arabidopsis thaliana*), the most similar protein to HvTIP2;2 was the maize protein ZmTIP2-3 (Besse et al. 2011). Several other monocot TIPs were present in the same clade: the maize TIPs ZmTIP2-1 and ZmTIP2-2, the rice TIP OsTIP2;1 and the barley TIP HvTIP2;1 (Besse et al. 2011). The genes encoding these TIPs are all mainly or solely expressed in roots (Chaumont et al. 2001; Lopez et al. 2004; Sakurai et al. 2005; Walley et al. 2016), with OsTIP2;1 known to be localised mainly in the stele and endodermis (Sakurai et al. 2008). Among the Arabidopsis TIPs, AtTIP2;2 and AtTIP2;3, were the most similar to HvTIP2;2 (Besse et al. 2011). The genes encoding AtTIP2;2 and AtTIP2;3 have both been shown to be expressed in the tonoplasts and central vacuoles of pericycle cells (Gattolin et al. 2009).

Although no aquaporin genes have been demonstrated to confer resistance against parasites or pathogens, there are reports of the involvement of TIPs in plant–nematode interactions. Transcriptomic analysis has shown that inoculation of Arabidopsis plants with either the beet cyst nematode *H. schachtii* or the root knot nematode *Meloidogyne incognita* affects TIP expression (Barcala et al. 2010; Szakasits et al. 2009). Similarly, a root-specific aquaporin, RB7, has been found to be upregulated during infection of transgenic tobacco plants with root knot nematodes (*M. incognita*, *M. arenaria* and *M. javanica*) (Opperman et al. 1994). Furthermore, a tomato TIP has been shown to interact with the *M. incognita* effector protein 8D05 until up to 24 days after inoculation (Xue et al. 2013).

While the main function of aquaporins is to facilitate water transport through membranes, an Arabidopsis TIP (AtTIP2;3) and a wheat TIP (TaTIP2;2) have also been found to transport NH_3_ (Bertl and Kaldenhoff 2007; Loqué et al. 2005). In Arabidopsis roots, the presence of ammonia increased the expression of AtTIP2;3 (Loqué et al. 2005). If the ability of HvTIP2;2 to transport water, NH_3_ or other compounds is enhanced in CCN-resistant barley plants, this might help explain the enlargement of vacuoles that has been observed in the syncytia of CCN-infected resistant plants (e.g. Aditya et al. 2015).

In the course of the fine-mapping research that is reported here, many new SNPs were discovered and assayed on resistant and susceptible materials. For any of these SNPs to be useful in marker-assisted breeding for CCN resistance, they should be diagnostic across a broad range of germplasm. Often, markers that are closely associated with traits in individual mapping populations prove to be unsuitable for marker-assisted selection in other cross combinations because marker alleles associated with the favourable trait are common even among materials that do not exhibit the desired trait. This is particularly true when mapping is conducted using arrays of previously discovered polymorphisms, given that common variants are generally preferred for the construction of such arrays. Here, the use of GBS for de novo SNP discovery provided an opportunity to discover new variants that might be specific to resistant materials. The inclusion of a range of susceptible cultivars in the GBS experiment provided an opportunity for early selection of potentially diagnostic SNPs. From the GBS data alone, four SNPs stood out because they distinguished the parents and progeny with *Rha2* resistance from susceptible parents and progeny and from cultivars with *Rha4* resistance. As fine mapping continued, KASP assays designed for these SNPs (wri297, wri321, wri322 and wri326) proved to be valuable in selecting resistant progeny for backcrossing. Across a larger panel of barley cultivars, assays wri321 and wri297 were both diagnostic of *Rha2*-based resistance. These markers have been readily adopted in commercial barley breeding in Australia, reducing reliance on costly phenotyping. Given that these two markers are particularly useful for marker-assisted selection, gel-based assays (wri328 and wri329) were developed to provide alternative ways to assay the same SNPs. These assays use temperature-switch PCR technology (Tabone et al. 2009), with which length polymorphisms can be generated from SNPs.

In conclusion, the research reported here narrowed the candidate region for the *Rha2* resistance gene to just 978 kbp and provided KASP and gel-based assays for each of two apparently diagnostic SNPs within that region. Evaluation of predicted genes within the candidate region revealed four genes that are known to be expressed in young roots. While three of these are discussed here as plausible candidates for *Rha2*, other possibilities cannot be excluded. Given that the barley reference genome sequence was assembled based on sequences from non-*Rha2* materials, it is also possible that the causal gene is not represented in the assembly. Further, if the expression of the causal gene is induced by infection, its expression would not be reflected in the BARLEX database and the gene itself might not be annotated as a high-confidence gene.

## Electronic supplementary material

Below is the link to the electronic supplementary material.

Supplementary material 1 (XLSX 353 kb)

Supplementary material 2 (PDF 1332 kb)

Supplementary material 3 (PDF 70 kb)
