# Supplementary material for: Fine mapping of Rha2 in barley reveals candidate genes for resistance against cereal cyst nematode
Source: Theor Appl Genet. 2019 Jan 18;132(5):1309–20. doi: 10.1007/s00122-019-03279-3 (PMC6476833; doi:10.1007/s00122-019-03279-3)
Supplement: Supplementary file 2 — Supplementary material 2 (PDF 1332 kb) [file 122_2019_3279_MOESM2_ESM.pdf]

Online Resource 2: Supplementary Figures

Fine mapping of *Rha2* in barley reveals candidate genes for resistance against cereal cyst nematode
Theoretical and Applied Genetics

Authors: Bart Van Gansbeke, Kelvin H.P. Khoo, John G. Lewis, Kenneth J. Chalmers and Diane E. Mather

Corresponding author: Diane E. Mather, School of Agriculture, Food and Wine, Waite Research Institute,
The University of Adelaide; diane.mather@adelaide.edu.au

Part 1 Tube tests for evaluation of resistance of cereal plants against the cereal cyst nematode *Heterodera avenae*

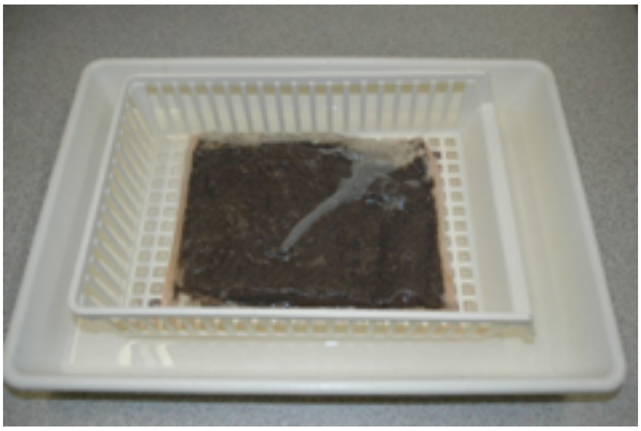

Fig. S1 A nematode 'farm' used as a source of larvae. A mixture of organic material and cyst-infested soil is packed in silk cloth and submerged in water. The farm is incubated at 5 °C in darkness. Eggs hatch within the cysts and J2-stage larvae emerge. A farm can be maintained for several months and larvae can be collected as needed

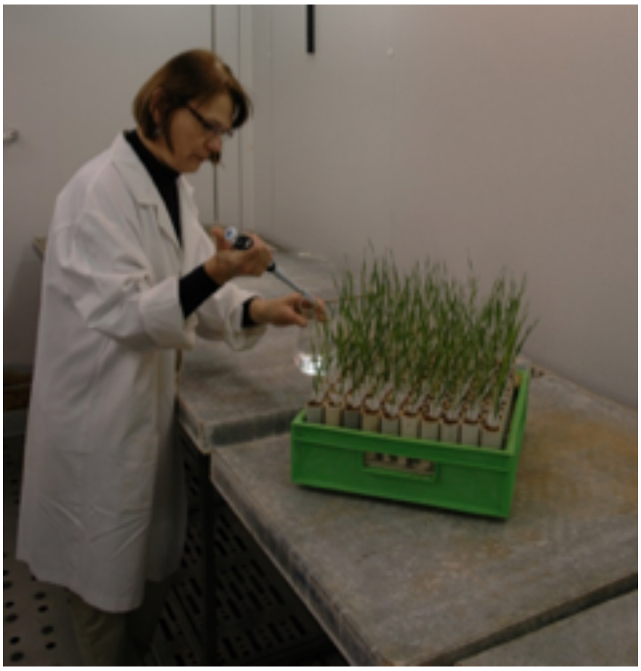

Fig. S2 Plastic tubes are filled with pre-sterilised sandy loam soil. One pre-germinated seed is sown in each tube. Tubes are arranged in a crate (10 rows x 10 columns). Aliquots of inoculum containing approximately 100 J2-stage larvae are pipetted onto the soil surface 1, 4, 7, 11 and 14 d later

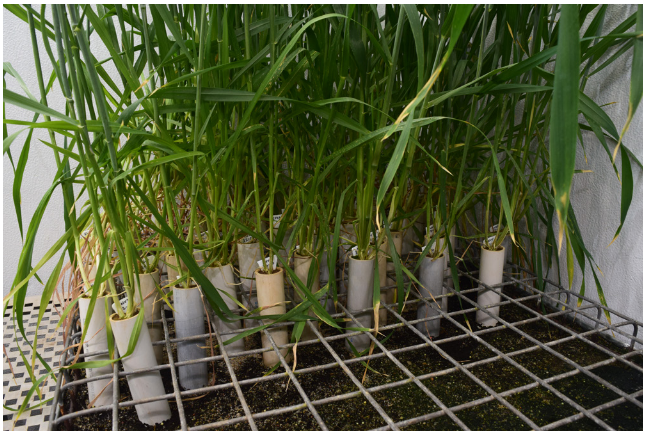

Fig. S3 After the final inoculation, tubes are arranged within a wire grid on a basal layer of cocopeat mixture

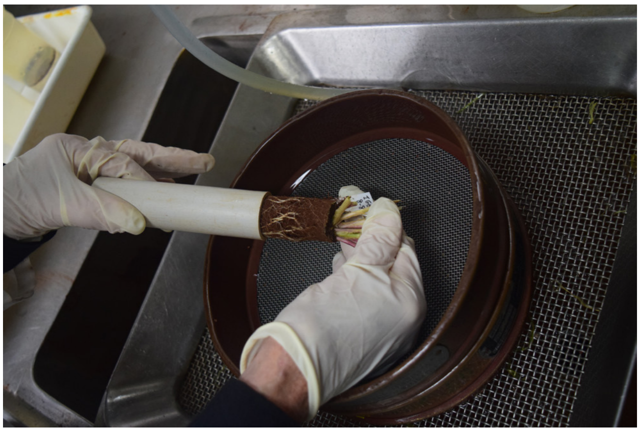

Fig. S4 At 70 d after the final inoculation, shoots are trimmed off. Tubes are removed from the grid. Roots and soil are removed fom each tube and thoroughly washed with a jet of water over a set of sieves, with the bottom sieve having apertures of 0.25 mm

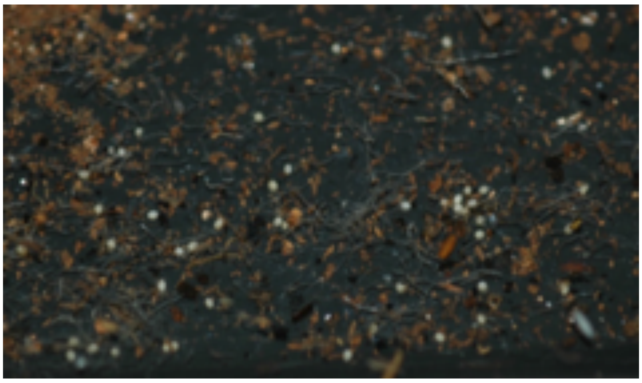

Fig. S5 Materials from the bottom sieve are spread out on a black plate and white cysts are counted. The root system is also examined for any white cysts remaining on the roots

Part 2 Pots tests for evaluation of resistance of cereal plants against the cereal cyst nematode *Heterodera avenae*

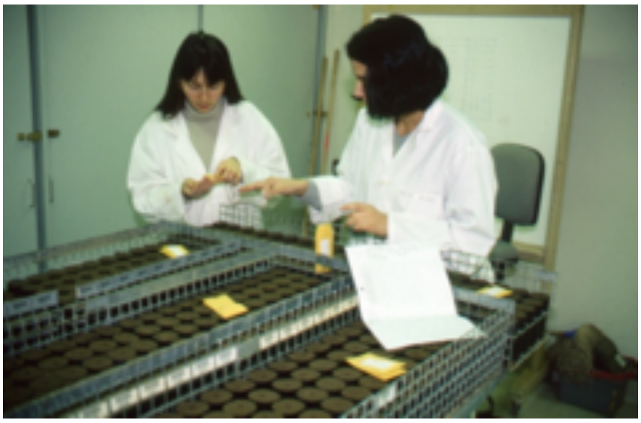

Fig. S6 Pots are filled with soil infested with brown cysts (approximately 25 eggs per g of soil) and arranged in wire mesh crates. One seed is sown per pot

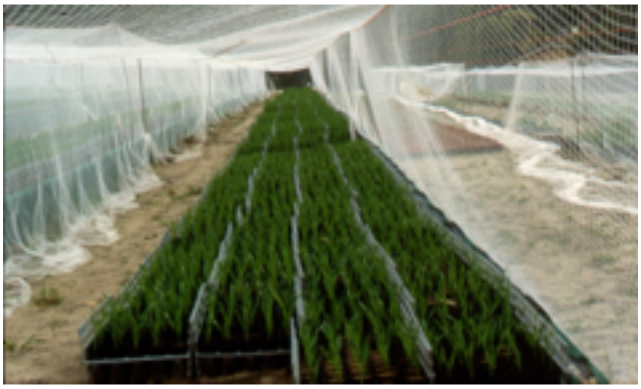

Fig. S7 Crates containing 50 plants each are placed outdoors in autumn, on well-drained terraces. Sprinkler irrigation is provided three times daily. Nets provide protection against birds

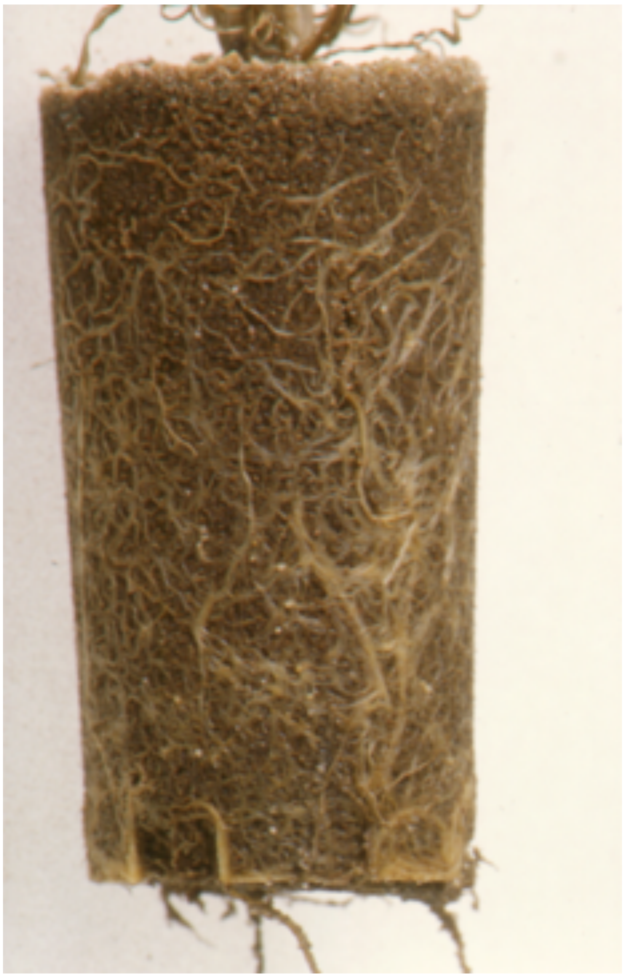

Fig. S8 After about 3 months, the root ball is removed from each pot. White cysts that are visible on the surface are counted

---

Part 3 Temperature-switch PCR markers

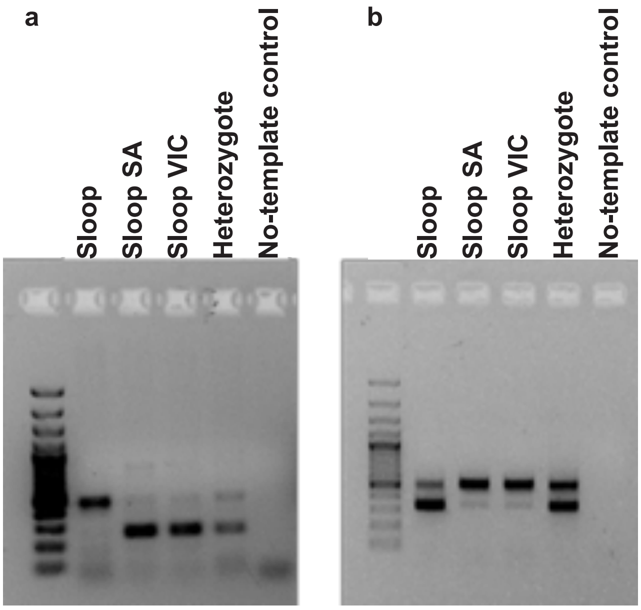

Fig. S9 Agarose gels showing products amplified by temperature-switch PCR from genomic DNA of Sloop, Sloop SA, Sloop VIC, a Sloop/Sloop SA heterozygote and a water control assessed with (a) the wri328 primer set, which amplifies a 457-bp product from the susceptible cultivar Sloop, a 250-bp product from the resistant cultivars Sloop SA and Sloop VIC and both products from heterozygotes; and (b) the wri329 primer set, which amplifies a 335-bp product from Sloop, a 514-bp product from Sloop SA and Sloop VIC and both products from heterozygotes
