# Supplementary material for: Fine mapping of Rha2 in barley reveals candidate genes for resistance against cereal cyst nematode
Source: Theor Appl Genet. 2019 Jan 18;132(5):1309–20. doi: 10.1007/s00122-019-03279-3 (PMC6476833; doi:10.1007/s00122-019-03279-3)
Supplement: Supplementary file 3 — Supplementary material 3 (PDF 70 kb) [file 122_2019_3279_MOESM3_ESM.pdf]

Online Resource 3: Supplementary File

Fine mapping of *Rha2* in barley reveals candidate genes for resistance against cereal cyst nematode

Theoretical and Applied Genetics

Authors: Bart Van Gansbeke, Kelvin H.P. Khoo, John G. Lewis, Kenneth J. Chalmers and Diane E. Mather

Corresponding author: Diane E. Mather, School of Agriculture, Food and Wine, Waite Research Institute, The University of Adelaide; diane.mather@adelaide.edu.au

---

Protocol for extraction of DNA from freeze-dried cereal leaf and endosperm tissues

Leaf sample tissue collection and preparation:

1. Approximately 2 cm^2 of leaf tissue was harvested from each seedling (at two leaf stage) and placed into 1.1 mL collection mini tubes (Axygen Scientific, California, USA)
2. The leaf samples were frozen and stored at -80 °C until freeze-drying commenced

Seed endosperm sample tissue collection and preparation:

1. Each seed was cut in two using a scalpel. The embryo-containing portion was stored at 4 °C for future use. The other (endosperm) portion was placed into a 1.1 mL collection mini tube (Axygen Scientific)
2. Prior to commencing freeze-drying, 75 µL of sterile water was added to each tube and the samples were kept at room temperature for 4 h
3. A 3 mm stainless steel ball bearing was added to each tube and the tubes were capped
4. The tissue was milled using a MM300 Retsch mill (Retsch GmbH, Germany) for 1 min at a frequency of 28.5 oscillations per second
5. The milled samples were frozen and stored at -80 °C until freeze-drying commenced

Freeze-drying

Frozen tissue samples were freeze-dried in an Alpha 1-2 LD freeze-dryer (Martin Christ Gefriertrocknungsanlagen GmbH, Germany) for 16 h. Conditions used were -50 °C and 110 mbar.

DNA extraction from freeze-dried leaf and endosperm samples:

1. Freeze-dried samples were milled using a MM300 Retsch mill (Retsch GmbH) at a frequency of 28.5 oscillations per second for 1 min
2. 600 µL of extraction buffer (0.1 M Tris-HCl, 0.05 M EDTA, 1.25% SDS (w/v)) was added to each sample. The samples were capped and shaken vigorously to resuspend the crushed tissue. Ball bearings were removed from each tube using a magnet and the samples were incubated at 65 °C for 30 min
3. Samples were cooled at 4 °C for 15 min then 300 µL of 6 M ammonium acetate (pre-chilled to 4 °C) was added to each sample. Samples were capped, shaken vigorously to ensure thorough mixing and incubated at 4°C for 15 min

4. Samples were centrifuged at 2570 rcf for 15 min to pellet down precipitated proteins and cellular debris
5. 600 μL of the supernatant from each sample was transferred to a new collection microtube containing 360 μL of isopropanol. Samples were capped, inverted 4-5 times to ensure thorough mixing and incubated at room temperature for 5 min to precipitate the DNA
6. Samples were centrifuged at 2570 rcf for 15 min to pellet down the DNA and the supernatant was carefully decanted. Remaining supernatant was allowed to drain off by inverting the sample tubes carefully and standing them on paper towel for 1 min
7. 400 μL of 70% ethanol (v/v) was added to each sample and the DNA pellet was dislodged by gently inverting the capped tubes once
8. Samples were centrifuged at 2570 rcf for 15 min and the supernatant was discarded. Samples were then incubated at 50 °C for 5 min to drive off residual ethanol
9. The extracted DNA samples were dissolved by resuspending the pellets in 200 μL of RNase A buffer (4μg/mL of RNase A in sterile water)
10. Samples were incubated at 4 °C overnight
11. The next morning, undissolved debris was pelleted down by centrifuging the samples at 2570 rcf for 20 min
12. 150 μL of the supernatant containing the extracted DNA was carefully transferred into fresh tubes and stored at -20 °C until required for downstream experiments
